# Probiotics for the Prevention of Acute Respiratory-Tract Infections in Older People: Systematic Review

**DOI:** 10.3390/healthcare9060690

**Published:** 2021-06-07

**Authors:** Maja Strauss, Dušanka Mičetić-Turk, Maja Šikić Pogačar, Sabina Fijan

**Affiliations:** 1 Faculty of Health Sciences, Institute for Health and Nutrition, University of Maribor, Žitna ulica 15, 2000 Maribor, Slovenia; maja.strauss@um.si (M.S.); dusanka.turk@um.si (D.M.-T.); 2 Faculty of Medicine, University of Maribor, Taborska ulica 8, 2000 Maribor, Slovenia; dusanka.micetic@um.si

**Keywords:** probiotics, fermented foods, upper respiratory tract infections, older people

## Abstract

The aim of this systematic review was to present the indirect influence of probiotics on the incidence and duration of acute upper respiratory-tract infections in older people, by regulating the immune system. Eight randomized, placebo-controlled clinical trials met the inclusion criteria, considering the threshold of older people being 60 years and over. Single strain probiotics were used in all studies, including three probiotic strains used in fermented foods: *Lactobacillus delbrueckii* subsp. *bulgaricus* OLL1073R-1, *Lacticaseibacillus paracasei* subsp*. paracasei* CNCM I-1518 and *Lacticaseibacillus* *paracasei* Shirota, and three probiotic strains used as food supplements: *Loigolactobacillus coryniformis* K8 CECT5711, *Bacillus subtilis* CU1 and *Lacticaseibacillus rhamnosus* GG. Current evidence showed that certain probiotic strains were better than a placebo in lowering the incidence or number of older people experiencing acute upper respiratory tract infections; however, not all probiotic strains were efficient, and not all studies reported statistically significant outcomes. More high quality large-scale properly controlled clinical studies focusing on older people are warranted.

## 1. Introduction

According to the consensus statement of the International Scientific Association for Probiotics and Prebiotics, probiotics are defined as “live microorganisms that, when administered in adequate amounts, confer a health benefit on the host” [1,2,3]. The most common probiotics are members of the *Lactobacillus* group, which has recently been divided into 25 genera [4] (including, but not limited to, certain strains of the following species: *Lactobacillus acidophilus*, *Lactobacillus delbrueckii* subsp*. bulgaricus, Lactobacillus gasseri, Lacticaseibacillus rhamnosus*, *Lacticaseibacillus casei*, *Lactiplantibacillus plantarum* and others), and *Bifidobacterium* genera (e.g., *Bifidobacterium infantis*, *Bifidobacterium longum* and others). Furthermore, certain strains from other bacterial species (e.g., *Lactococcus lactis*, *Enterococcus faecium*, *Streptococcus thermophilus*, *Bacillus subtilis*, *Escherichia coli*) and even certain strains from certain yeasts (e.g., *Saccharomyces cerevisiae* var. *boulardii*) qualify as probiotics [5]. Scientific evidence of probiotic benefits on human health is continuously expanding, and there are enough data to justify investigation of probiotics for treatment or prevention of several disorders, from antibiotic and *Clostridium difficile*-associated diarrhea, irritable bowel syndrome and inflammatory bowel disease, to anxiety, depression and wound healing [6,7,8,9,10].

Another consensus statement of the International Scientific Association for Probiotics and Prebiotics reported the definition of fermented foods as “foods made through desired microbial growth and enzymatic conversions of food components” [11]. Although fermented foods have been consumed for thousands of years, they have been receiving increased attention among biologists, nutritionists, technologists, clinicians and consumers, as research has shown that fermented foods could improve gastrointestinal and systemic health [11,12,13,14,15]. Delivery of probiotics through fermentation [16] is a synergistic approach that uses the positive effects of both fermented foods and probiotics.

Acute upper respiratory tract infections (URTIs) are illnesses caused by infection of mucosal surfaces in the nose, sinuses, pharynx and/or larynx and large airways. These infections include non-allergic rhinitis (the common cold), acute sinusitis, acute pharyngitis, tonsillitis acute laryngitis, acute epiglottitis (supraglottitis) and acute otitis media [17,18,19]. URTIs can be caused by viruses or bacteria. Common respiratory viruses include influenza viruses, respiratory syncytial viruses, parainfluenza viruses, rhinoviruses and enteroviruses, adenoviruses, coronaviruses and others [18,20,21,22]. Common bacterial pathogens causing URTIs are *Streptococcus pneumoniae, Streptococcus pyogenes*, *Haemophilus influenzae*, *Moraxella catarrhalis*, *Mycoplasma pneumoniae* and others [19,20,23]. URTIs are a very common problem among infants, children and elderly, and they account for 9% of all consultations in general practice [17,20]. Upper respiratory tract infections (URTI) are one of the most common acute infections in the outpatient setting, causing more outpatient doctor and emergency care visits and higher antibiotic use, even though several common acute infections, such as colds and influenza, are viral illnesses, where antibiotic use is inappropriate [18,24,25,26].

A large pool of evidence in well-designed reviews has suggested that probiotic supplementation reduces episodes of common infectious diseases, including respiratory tract infections, through improvement of immune function [17,27,28,29,30,31,32,33]. Several clinical studies have found that probiotic supplementation can reduce the duration of symptoms in otherwise healthy children and adults with common acute respiratory conditions [26,34,35,36,37]. The Cochrane library, which is a global, well-known collection of high-quality, independent evidence database, including high-quality reviews to inform healthcare decision-making, includes a review by Hao and co-authors [17], which also suggested focusing on older people or performing a subgroup analysis of older people.

Life expectancy has been increasing in the last decades. However, the process of ageing is associated with a decline of many functions, increasing frequency of chronic diseases or age-related disease, such as atherosclerosis, Alzheimer’s dementia, diabetes mellitus and osteoporosis, impaired mobility and decreased cognitive functions. It has also been found that human immune function undergoes adverse changes with ageing. This immune senescence potentially leads to an increased risk of infections and certain cancers in the elderly. The reasons for this include epidemiological elements, immunosenescence and malnutrition, as well as a large number of age-associated physiological and anatomical alterations, including physiological changes in the diversity and loss of resilience of the intestinal microbiota, leading to more permissive communication along the gut–lung axis [17,38,39,40,41]. This is also the reason that acute respiratory infections are common among nursing home residents, representing about one-third of all infections, and they are the most frequent reason for hospital admittance and a significant cause of mortality [42,43].

The most common threshold reported in literature for determining the elderly population (also referred to as older people) with regards to these physiologies is 65, although some elderly are as vital even at 75 years, leading to much debate on whether to increase the age of the population determined as older people [44,45]. The categorization of the age of older people being 65 years and over and oldest-old being 80 years and over is also in line with recommendations of the World Health Organisation, whilst the United Nations defines an older person as a person over 60 years of age [46] and provides figures for both 60 years and 65 years and older [47], although literature also suggests redefining older people based not on chronical age or generic definitions, but focusing more on establishing a direct link between an individual patient’s characteristics [45].

The aim of this review was to determine the efficiency of probiotic consumption, either as food supplements or part of fermented foods, on the incidence and duration of upper respiratory tract infections in older people.

## 2. Materials and Methods

### 2.1. The Literature Selection Process

The systematic review was conducted using the guidelines of the PRISMA (Preferred Reporting Items for Systematic Reviews and Meta-Analyses) statement. The research strategy was developed using the PICOS (Population, Intervention, Comparator, Outcomes, Study type) framework (Table 1) [48].

As noted in Table 1, the inclusion criteria included that (1) the study population were older people aged 60 and over according to the UN tabulations; (2) probiotics were supplemented either in fermented drinks/foods or as capsules; (3) the study trial was randomized, controlled; (4) incidence and/or duration of upper respiratory tract infections (URTI) was reported. The exclusion criteria included that (1) the participants or the study population were children, adults younger than 60 years, older people as undetermined part of adults, animal studies or in vitro studies; (2) heat-killed “probiotic” supplements or fermented foods without added probiotics were used; (3) the control was another probiotic (4) upper respiratory tract infections were not reported or only reported as an undetermined part of common infectious diseases (CID) that can also include gastrointestinal infections; (5) study design was non-randomized and non-controlled clinical trials, secondary research study (reviews or meta-analyses), study was published as conference proceeding or editorial or only the abstract was available. The literature search was conducted in December and followed-up in January 2021. The following databases were searched: Pubmed (n = 5), ScienceDirect (n = 31), Web of Science (n = 3), EBSCOhost (n = 8), Scopus (n = 2), Manual search (n = 61), with the total in January 2021 (n = 110) using the search strategy: (elderly OR “older people” OR “older adult” OR senior) AND (probiotic OR “probiotic fermented”) AND (“respiratory tract infections” OR RTI) NOT children. The asterisk was used to allow for flexibility on the word ending, e.g., probiotic* would have identified “probiotic” and “probiotics”; elder* would have identified “elderly” and “elder people” [27]. For an article to be identified, the search terms from the keywords were required to appear either in the title or abstract of the articles; however, some database search engines do not search for keywords in the main text, leading to two articles being included from the reference lists of included studies. The literature search was conducted independently by MS and SF. If inconsistencies occurred, both authors discussed the differences until a consensus was reached.

### 2.2. Data Extraction and Assessment of Selected Clinical Trials

Data extraction included study setting, study design, timeline of intervention and observation period, number of enrolments, number of participants that completed the study, probiotic intervention, information on placebo, dosage and duration of intervention, investigated outcomes, observations and adverse events, where applicable. The Joanna Briggs Institute critical appraisal tool for randomized controlled trials was used to assess the quality of included studies [49]. Study quality was rated individually by MS and SF.

## 3. Results

**Figure 1:**
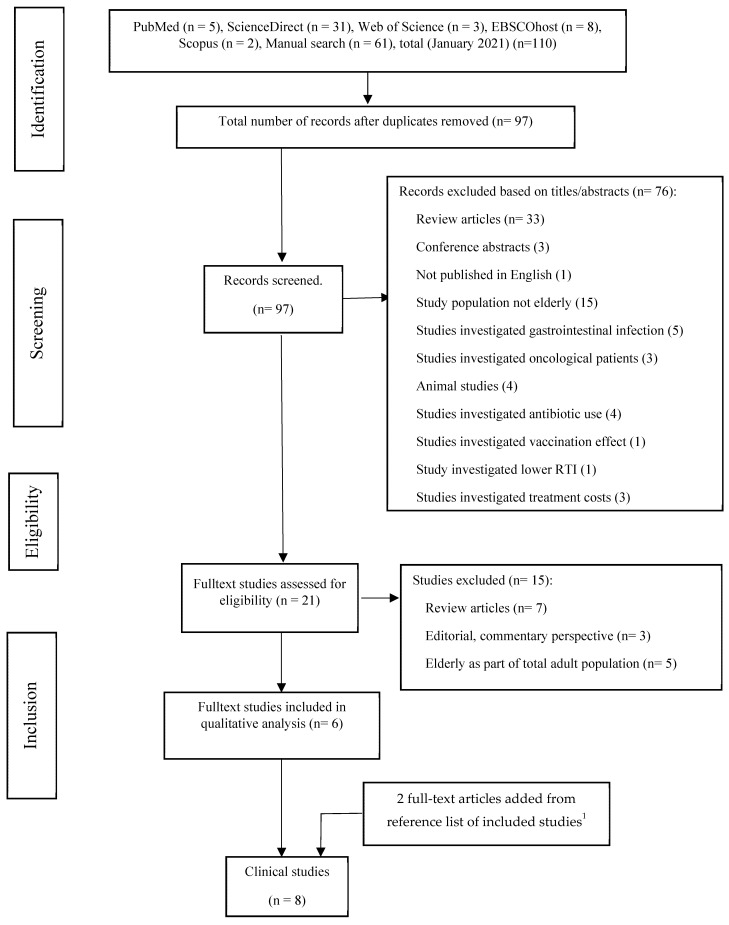
Flow chart of literature search procedure according to the PRISMA statement [48]. ^1^ two references identified from the reference lists [38,53,54].

The literature search yielded 110 titles, of which six studies met all inclusion criteria and none of the exclusion criteria [38,50,51,52,53,54]. Two additional studies [39,55] were found from the reference lists of the included studies of [38,53,54], yielding a total of eight studies included in the review. The PRISMA flow diagram of the literature search is noted in Figure 1.

The study [39] was additionally found in the reference list of included studies [38,53,54] and did not contain the phrase probiotic in its title. On the other hand, the study [55] contained the phrase probiotic in its title, but contained ‘winter infections’ and ‘respiratory diseases’ (ear, nose and throat or ENT), instead of ‘’upper respiratory tract infections’. These are perhaps the reasons they were not found using the literature search strategy.

At first, 10 studies seemed to meet the inclusion criteria. However, after careful review of the full-text, we found that two of these studies [36,56] were conducted on the same study group of adults (18 to 67 years, mean age 36 ± 12 years), but they were not elderly, according to our threshold. Despite this, we excluded both studies and also two additional; therefore, a total of four studies [35,36,37,56] were excluded due to a different definitions of elderly below our threshold of 60 years. The study [35] defined elderly as ≥45 years (mean age of the intervention group was 57.39 ± 8.47 years, mean age of control group was 59.54 ± 8.08 years). In the study [37], subjects were considered elderly at 50 years, as the working population is entitled to early retirement in Malaysia. Although the mean age of the intervention group was 60.6 ± 6.5 years and the mean age of control group was 63.6 ± 8.3 years, we decided to exclude this study from our review, as this group consisted of participants aged 50 years and older, thus not meeting the threshold of 60 years.

The methodology of the studies was also assessed using the Joanna Briggs Institute critical appraisal tool (JBI) for randomized controlled trials, noted in Table 2.

Four of the investigated 8 studies’ (Fonollá, Fujita, Guillemard, Van Puyenbroeck) study quality was rated as good, as all 13 questions of the JBI checklist for RCTs were answered as ‘yes’ [38,50,51,53].

In the study by Lefevre [52], the clinical outcomes were conducted on the subset of the first 44 subjects; thus, the outcomes were not measured in the same way for all treatment groups. It is also unclear how they determined the allocation to each group in this subset, as exactly 22 are in each group; thus, perhaps individual randomization and parallel group formation was not appropriate; therefore, we rated questions 10 and 13 as NO.

The Wang study noted its design as double-blind, but no blinding procedure was noted, and there was no information regarding the possible similar appearance of intervention and control; therefore, we rated questions 2, 4 and 5 as NO [54].

The Turchet and Makino studies [39,55], on the other hand, had several limitations, and we rated them as poor; however, we decided to include them in our review, as we wished to focus all possible studies on the incidence or duration of URTI for older people. The Turchet study was open-labelled, and the control group did not receive any placebo; therefore, the study was not blinded. The authors also did not explain their randomization process. On the other hand, the outcomes were measured in all groups, and data was adequately analyzed. The Makino study did not report the randomization procedures in the methods section, but only mentioned randomization in the results. This study used milk as a control but did not state if the appearance of the intervention and control differed; thus, it was not possible to determine if the study was blinded. However, the outcomes were also measured in all groups, and data was also adequately analyzed.

In the Cochrane review on probiotics for preventing acute upper respiratory tract infections of all population groups [17], the authors decided to omit the Guillemard and Turchet studies since the participants were vaccinated against influenza; however, in our review, we focused only on the elderly and decided to include these two studies, as it is a real possibility for the elderly to receive an annual influenza vaccination. Another review study [27] also included the Guillemard study in their review of the efficacy of *Lacticaseibacillus paracasei* subsp. *paracasei* CNCM I-1518 on common infectious diseases in all populations.

Due to the taxonomic update of the genus *Lactobacillus* [4], the new nomenclature of the probiotic strains from the lactobacilli genera used in the clinical studies are noted in Table 3. Commercial name: fermented drink Actimel (Danone).

The description of the eight clinical studies on the effects of fermented drinks with probiotics and probiotic food supplements against acute upper respiratory tract infections in older people is noted in Table 4, and the outcome of these studies are reported in Table 5.

All studies included in the review were randomized, controlled trials. Three studies were multicenter studies conducted in nursing homes [50,53,54], and one study was a multicenter study conducted in day-care facilities [51]. Four studies were conducted on free-living elderly individuals [38,39,52,55], recruited either from referential or general practitioner centers. Two studies on free-living elderly individuals did not specify the manner of recruitment of the free-living elderly [39,52]. Two studies were pilot studies [54,55]. The studies were conducted in Italy, Japan, France, Belgium, Spain and Canada, thus being distributed through three (Europe, America, Asia) of the six continents.

Five studies investigated the influence of fermented foods with probiotics [39,50,51,53,55]. Two of these studies [38,55] investigated the influence of a fermented drink with *Lacticaseibacillus paracasei subsp. paracasei* CNCM I-1518 (Actimel, Danone) and yoghurt cultures, two studies investigated the influence of a fermented milk with *Lacticaseibacillus* *casei* Shirota and lactic acid bacteria (Yakult) [51,53] and one study investigated the influence of yoghurt fermented with *Lactobacillus delbrueckii* subsp. *bulgaricus* OLL1073R-1 and yoghurt culture *Streptococcus thermophilus* OLS3059 [39]. Three further studies investigated the influence of supplementation with capsules containing single-strain probiotics: *Loigolactobacillus coryniformis* K8 CECT5711 [50], *Bacillus subtilis* CU1 [52] and *Lacticaseibacillus rhamnosus* GG [54]. The duration of intervention was also very different, ranging between six months [53,54], four months [51], three months [38], eight weeks [39], three weeks [55], 10 days intermittently, an alternating 18-day break repeated four times [52] to two weeks [50].

The studies differed in sample sizes, noted in descending order: 1072 study participants [38], 554 [53], 360 [55], 196 [54], 154 [51], 142 [39], 100 [52] to 84 participants [50].

The incidence of URTIs, or common colds or winter pathologies, namely influenza syndromes (respiratory diseases, ear, nose and throat (ENT) pathologies) after consumption of probiotics or control was investigated in all eight clinical trials [38,39,50,51,52,53,54,55] as shown in Table 3. Of these, five studies [38,39,50,51,52,54] reported a statistically significantly lower incidence or risk of URTIs or certain symptoms of URTIs. Four studies [38,51,53,55] also investigated the difference in duration of URTI after supplementation with probiotics or control. Of these, three reported [38,51,55] a significantly shorter duration of URTI (1–1.7 days) in the treatment group of between groups, whilst the study [53] did not report a significant difference between the groups. Two studies did not report any statistically significant difference of any investigated outcomes [53,54].

Immunological parameters were reported in four of the eight studies [38,39,50,52]. Two of these studies reported a significant increase of natural killer cells [39] and IFN-γ concentrations [52] in the treatment group compared to the control, whilst two studies reported no significant difference [38,50].

One study [50] reported an adverse event of gastrointestinal problems that could have been associated with analgesic consumption during the clinical trial. Another reported adverse events that occurred during the clinical trial, including 46 cases of dyspepsia [55] and one case of serious bronchopneumonia [55]. However, according to the authors, none of these adverse events were related to the consumption of probiotics or the control. Three studies [38,52,54] reported the number of all general health-related occurrences during the clinical trials as 416, 193 and 755, respectively. However, the authors of these studies noted that the number of events were similar in both the intervention and the control group and that none were found to be related to probiotic consumption. Three studies reported no adverse events related to probiotic consumption [39,51,53].

## 4. Discussion

In this review, we found that probiotics were better than a placebo in reducing the incidence or risk and duration of acute upper respiratory tract infection (URTI) in older people. Adverse events were minor and not related to probiotic consumption. The mode of action of probiotics was most probably systemic immunomodulation via interaction of the microorganisms with the mucosal immune system by various methods, including colonization resistance, trans-epithelial resistance, increased number and activity of natural killer cells, release of certain cytokines and bacteriocins, enhanced antibody response, stimulation of non-specific immunity, enhancing humoral and cellular immunity as well as co-mediation of metabolic and immune homeostasis [31,57], leading to better communication between the gut–lung axis [41].

Single strain probiotics were used in all studies, including three probiotic strains used in fermented foods (*Lacticaseibacillus paracasei* subsp*. paracasei* CNCM I-1518, *Lacticaseibacillus* *paracasei* YIT 9029 and *Lactobacillus delbrueckii* subsp. *bulgaricus* OLL1073R-1) and three probiotic strains used as food supplements (*Loigolactobacillus coryniformis* K8 CECT5711, *Bacillus subtilis* CU1 and *Lacticaseibacillus rhamnosus* ATCC 53103).

*Lacticaseibacillus paracasei* subsp*. paracasei* CNCM I-1518, formerly known as *Lactobacillus casei* DN-114 001, is the probiotic found in the fermented drink Actimel^®^ from Danone. It is a well-known probiotic with several beneficial health effects, including increased relevant specific antibody responses to influenza vaccination in individual over 70 years of age [58], reduced risk of common infections in stressed individuals, such as shift workers [59] and lowering the rate of common infectious diseases (CID) [60] and acute diarrhea in children [61]. Two studies in our review used this probiotic-containing fermented drink as the intervention [38,55], and both studies resulted in at least one reported statistically significant beneficial effect regarding URTIs for older people.

*Lacticaseibacillus paracasei* YIT 9029, more commonly known as *L. paracasei* Shirota, formerly *Lactobacillus casei* Shirota, is a well-known probiotic found in the fermented drink Yakult. Many clinical studies support its use as a probiotic, including several studies on older people, as the following health benefits have been found: decreasing the daily risk of infection and improving the quality of life among the residents of facilities for the elderly [62,63,64,65,66,67]. Two studies in our review used this probiotic-containing fermented drink as the intervention [51,53]; however, only the study by Fujita and co-authors reported statistically significant lower duration of URTI infection in the treatment group compared to the control group [51], whilst the study by van Puyenbroeck and co-authors reported no significant difference for the number of participants with respiratory symptoms or the number of days with respiratory symptoms of the treatment group compared of the control [53]. Although the intervention period was longer in the second study (six months compared to four months in the Fujita study), and the dosage was two drinks per day for the second study compared to the Fujita study, the study population in the van Puyenbroeck study was nursing home residents from Belgium with a median age of 84.17 years, whilst the participants in the Fujita study were Japanese users of day-care facilities with a mean age of 83.2 years. According to the World Bank data, the average life expectancy in 2019 was the highest at 84.9 years, and in Belgium, it was 81.6 years. Therefore, perhaps the Japanese day-care participants had better generic definitions and an immune system that was more susceptible to positive change due to probiotic intervention, whilst the nursing home residents in Belgium did not, thus meaning that chronological age is not the only factor to consider [45].

*Lactobacillus delbrueckii* subsp. *bulgaricus* OLL1073R-1, together the strain *Streptococcus thermophilus* OLS3059, is part of a yoghurt (1073R-1 yoghurt) produced in Japan by Meiji. *Lactobacillus* delbrueckii subsp. *bulgaricus* OLL1073R-1, a polysaccharide-producing lactic acid bacterial strain, has proven beneficial health effects on older people by preventing infection with influenza A virus subtype H3N2 via increasing the production of H3N2-bound salivary IgA [68] and improving the mucosal immune function in elderly people with weakened immune systems [69]. The study by Makino and co-authors [39], which was among the investigated studies in this review and was additionally found in the reference list of included studies [38,53,54], also used this probiotic. Since the title of the Makino study did not contain the word probiotic, it was not found in the literature search but was included subsequently. This study referred to the intervention as yoghurt fermented with *Lactobacillus delbrueckii* subsp. *bulgaricus* OLL1073R-1, and two statistically significant positive effects were observed: significantly lower risk of catching a cold and significantly higher increase of natural killer cell activity in the treatment group compared to the control group [39]. Other research has also revealed that this strain indeed has probiotic properties as noted in the large-scale properly controlled clinical trial conducted on 961 participants [70], where it was found that a statistically significant beneficial health effect was observed (increase in IFN-gamma). This is also in line with the definition that if a health benefit of a probiotic strain is indeed proven with a clinical study, it can be considered a probiotic strain [71], and the observation of similar health benefits for different strains of the same species in well-designed clinical studies justify it as a probiotic [72].

*Bacillus subtilis* CU1 is a recently described and patented probiotic strain [73]. In a recent clinical trial on healthy elderly subjects receiving 2 × 10^9^ spores of *Bacillus subtilis* CU1 per day for 40 days, it was found to be safe and well-tolerated in the clinical subjects without undesirable physiological effects on markers of liver and kidney function, complete blood counts, hemodynamic parameters and vital signs [74]. The beneficial effects of this strain on the immune health of free-living elderly subjects were also reported in the study by Lefevre and co-authors [52]. A significantly lower occurrence of infectious episodes associated with respiratory infections and a significant increase in concentrations of INF-gamma were observed in the treatment group compared to the control. This study found that systemic as well as intestinal and respiratory mucosal immune responses of older people was indicated by the increased concentrations of fecal and salivary secretory IgA and serum INF-gamma.

*Loigilactobacillus coryniformis* K8 CECT5711 (formerly *Lactobacillus coryniformis* K8 CECT5711) is a reuterin-producing probiotic strain that has been found to have immunomodulatory activity, in which fermented milk containing *Loigilactobacillus coryniformis* K8 CECT5711 in combination with the strain *Lactobacillus gasseri* CECT5711 was administered to healthy adults and enhanced both innate and specific immune responses, including an increase in the proportion of natural killer cells and IgA concentrations [75]. Two additional studies performed in children with the same fermented milk containing the combination of *Lactobacillus* strains corroborated this effect on the immune system [76,77]. One study by Fonollá and co-authors in our review used this probiotic as an intervention for nursing home residents [50]. A significantly lower incidence of respiratory symptoms (sore throat) and consumption of analgesics as well as a higher percentage of responders to the influenza vaccine in the treatment group compared to the control group were found.

*Lacticaseibacillus rhamnosus* ATCC 53103, more commonly known as *L. rhamnosus* GG or LGG (formerly *Lactobacillus rhamnosus* GG), was the first strain of the genus lactobacilli to be patented in 1989, and it is among the most characterized, utilized and studied probiotics [78]. It has many different health benefits, including producing a biofilm that mechanically protects mucosa, different soluble factors beneficial to the gut by enhancing intestinal crypt survival, diminishing apoptosis of the intestinal epithelium, preserving cytoskeletal integrity as well as pathogen inhibition, promoting immune responsiveness by reducing expression of inflammation markers and increasing production of IL-10, IL-12 and TNF-alfa [79]. Several clinical studies have been conducted with older people using *Lacticaseibacillus rhamnosus* GG, and they have reported positive results [80,81,82]. However several other clinical studies did not report any health benefits for LGG use [83,84,85]. One study in our review by Wang and coauthors used this probiotic as an intervention [54] but did not find a statistically significant difference in confirmed viral respiratory infections, influenza-like illness, hospitalization over pneumonia or other measured outcome between groups for nursing home residents, but only a lower risk reduction for influenza and other respiratory viral infections in the intervention group compared to the control. The authors noted that the study was a pilot study and therefore not powered to be statistically significant. They also noted that a large scale RCT is warranted.

An important aspect for probiotics and URTIs is the newly emerged coronavirus SARS-CoV-2 as a causative agent of respiratory-tract infections. The potential application of probiotics for the prevention and treatment of COVID-19 will probably be extensively investigated in the future, and several reviews also address the question of whether probiotics could be used as possible adjuvant therapy in the prophylaxis and/or alleviation of COVID-19 symptoms [66,86,87,88]. The main basis for this possibility is that several clinical studies have reported alterations of gut microbiota/dysbiosis of COVID-19 patients [89,90,91], and it is well-known that probiotics are efficient in positively modulating the gut microbiome in many cases of dysbiosis as has been noted in systematic reviews and meta-analyses [92,93,94,95]. To date, one clinical study has addressed this question, and it concluded that support for further trials to assess the potential role of probiotics in preventing viral URTI (and possibly also COVID-19) is warranted [96].

There are at least 150 published clinical trials that have assessed the beneficial effect of probiotic consumption in preventing URTIs conducted on various populations. Some positive effects on various groups of adults from recent studies include significantly fewer community-acquired colds [97], significantly decreased URTI symptoms [98], significantly reduced URTI [99] and significantly fewer number of days with URTI symptoms [100]. Another study [37] also found a statistically significant reduction in the duration of nasal, pharyngal and general flu symptoms as well as total respiratory illnesses via reduced plasma pro-inflammatory cytokines (Il-1) and increasing anti-inflammatory cytokines (Il-4, Il-10). However, it was excluded from our review due to the fact that the population in the study was considered elderly at 50 years, as the working population in Malaysia is entitled to early retirement, and thus, it did not comply with our threshold for older people at 60 years and older [46]. Recent studies have also found positive effects of probiotics against URTIs in children [101,102,103]. All these studies have shown that probiotics can be effective in alleviating URTI symptoms by shortening the duration as well as lowering the incidence of URTI in various populations.

One aspect that is an important factor in choosing a probiotic is the format. Probiotics can be sold as capsules, sachets, tablets or as part of fermented foods or drink [104,105]. Older people may have problems with swallowing tablets or whole capsules, which would require crushing/splitting tablets or opening capsules to facilitate probiotic administration [106], and they may prefer to prepare their probiotic drink by adding lyophilized probiotics in the form of granules from sachets into water. On the other hand, consuming a commercial probiotic-containing drink or yoghurt is very convenient for older people as it is easy and something familiar, and it does not seem like they are taking yet another pill or tablet. The positive effects of fermented drinks or yoghurts with added probiotics are not only due to the added probiotic but are also attributed to the ferments or metabolites resulting from the proprietary fermentation process from the starter cultures used in yoghurt fermentation, most commonly *Streptococcus thermophilus* and *Lactobacillus bulgaricus* [58]. Furthermore, the incorporation of probiotics into dairy foods may aid in tolerating harsh gastro-intestinal conditions better than those of non-dairy carrier foods, as the buffering action of milk as well as milk fat might protect probiotics in such conditions by reducing their direct exposure to harsh conditions [16,105,107]. In our review, both probiotics in fermented foods and as food supplements were mainly effective.

Probiotic strain selection is a very important step in conducting clinical trials, and most decisions for strain selections in clinical studies are based on positive results of previous trials as well as the support of manufacturers. In our reviewed studies, the manufacturers were either noted as co-authors [38,39,50,52,55], with statements regarding conflicts of interest, or were noted in the section “conflict of interest” as a partial funder of research [54] or as a supporter of research within affiliation information [51,53]. Most clinical studies have assessed commercial probiotics or probiotic-enriched foods, and it is important to address the careful product characterization and potency assessment and reporting in clinical trials by accurate declaration and actual concentrations of probiotic strains and rigorously using newly available molecular tools [108]. This is also in line with the definition of probiotics as being exactly defined and consumed in adequate amounts [1]. The manufacturers may also account for the burden of proof and safety, GRAS (generally regarded as safe) process complying with FDA approval, where applicable, and other processes depending on the country policies and the classification of the used probiotic as a drug, functional food or food supplement [108]. All of this involves an active role of the manufacturer, and therefore, co-authorship of researchers from the manufacturer could be justified, provided the results are reported objectively.

## 5. Conclusions

Current evidence has shown that certain probiotic strains, as probiotics in food supplements and as part of dairy fermented foods, are better than a placebo, as most investigated clinical trials exhibited a positive health benefit for older people regarding incidence and/or duration of upper respiratory tract diseases and/or immune modulation, which is in favor of reducing URTIs, as most studies reported statistically significant differences; however, the studies were diverse in intervention duration, chosen probiotic and determination of primary outcome. In light of this, the fact that URTIs represent important infections among older people and the well-established immunomodulatory role of probiotics mean that even a minimal reduction in the incidence, risk or duration of URTIs in older people would have an important clinical and economic impact. Therefore, more high-quality, large-scale, properly controlled clinical studies focusing on older people are warranted.

Future RCTs should be designed to assess outcomes commonly reported in other clinical studies to enable comparison, and they should implement longer intervention times to enable modulation of the immune system of the elderly. Future studies should also incorporate adequate description of blinding and concealment of the allocation sequence and a more detailed report of adverse effects, and they should not be unduly influenced by manufacturer funding.

## Figures and Tables

**Table 1:** Study design according to PICOS framework (population, intervention, comparator outcome, study type).

Frame	Inclusion Criteria	Exclusion Criteria	Search Terms ^1^
Population	Older people, over 60 years	Children, adults younger than 60 years or older people as undetermined part of adults	elder* OR “older people” OR “older adult” OR senior
Intervention	Probiotic fermented foods/drinks or probiotic food supplements	Heat-killed “probiotic” supplements, fermented foods without added probiotics	probiotic* OR “probiotic fermented”
Comparison	Control group (can be placebo, prebiotic or non)	Another probiotic	/
Outcome	Incidence and/or duration of upper respiratory tract infections (URTI)	Upper respiratory tract infections not reported or only reported as undetermined part of common infectious disease that can also include gastrointestinal infections,	“respiratory tract infections” OR RTI,
Study type	Randomised, placebo controlled clinical trials	Non-randomised, non-controlled clinical trials. Reviews and meta-analyses. Conference proceedings, editorial letters, only abstract available.	^2^

^1^ Final search strategy: (elderly OR “older people” OR “older adult” OR senior) AND (probiotic OR “probiotic fermented”) AND (“respiratory tract infections” OR RTI) NOT children; ^2^ Manual removal of article, if relevant.

**Table 2:** Quality assessment checklist of included clinical trials using the Joanna Briggs Institute critical appraisal tool for randomized controlled trials.

First Author, Year	1	2	3	4	5	6	7	8	9	10	11	12	13
Turchet 2003 [55]	unclear	no	yes	no	no	no	yes	yes	yes	yes	yes	yes	no
Makino 2010 [39]	unclear	yes	yes	unclear	unclear	yes	yes	yes	yes	yes	yes	yes	unclear
Guillemard 2010a [38]	yes	yes	yes	yes	yes	yes	yes	yes	yes	yes	yes	yes	yes
Fujita 2013 [51]	yes	yes	yes	yes	yes	yes	yes	yes	yes	yes	yes	yes	yes
Puyenbroeck 2012 [53]	yes	yes	yes	yes	yes	yes	yes	yes	yes	yes	yes	yes	yes
Fonolla 2019 [50]	yes	yes	yes	yes	yes	yes	yes	yes	yes	yes	yes	yes	yes
Lefevre 2015 [52]	yes	yes	yes	yes	yes	yes	yes	yes	yes	no	yes	yes	no
Wang 2018 [54]	yes	no	yes	no	no	yes	yes	yes	yes	yes	yes	yes	yes

Note: possible answers: YES, NO, Unclear, not applicable (NA); 1. Was true randomization used for assignment of participants to treatment groups? 2. Was allocation to treatment groups concealed? 3. Were treatment groups similar at the baseline? 4. Were participants blind to treatment assignment? 5. Were those delivering treatment blind to treatment assignment? 6. Were outcomes assessors blind to treatment assignment? 7. Were treatment groups treated identically other than the intervention of interest? 8. Was follow up complete and if not, were differences between groups in terms of their follow up adequately described and analyzed? 9. Were participants analyzed in the groups to which they were randomized? 10. Were outcomes measured in the same way for treatment groups? 11. Were outcomes measured in a reliable way? 12. Was appropriate statistical analysis used? 13. Was the trial design appropriate, and were any deviations from the standard RCT design (individual randomization, parallel groups) accounted for in the conduct and analysis of the trial?

**Table 3:** Commercial names, manufacturing information of used probiotics and updated names according to new taxonomic nota.

Study	Manufacturing Information	Name of Probiotic Strain, Mentioned in Study	Name according to New Taxonomic Note ^1^ or Otherwise Updated Name	Abbreviation
Turchet 2003 [55]	Danone (France)	*Lactobacillus casei* DN-114 001	*Lacticaseibacillus paracasei* subsp*. paracasei* CNCM I-1518	LpCNCM
Makino 2010 [39]	Meiji Dairies cooperation (Japan)	*Lactobacillus delbrueckii* subsp. *bulgaricus* OLL1073R-1	unchanged	LbR-1
Guillemard 2010a [38]	Danone (France)	*Lactobacillus casei* DN-114 001	*Lacticaseibacillus paracasei* subsp*. paracasei* CNCM I-1518	LpCNCM
Fujita 2013 [51]	Yakult Honsha (Japan)	*Lactobacilluscasei* Shirota (*Lactobacillus* *casei* YIT 9029)	*Lacticaseibacillusparacasei* Shirota	LcS
Puyenbroeck 2012 [53]	Yakult Honsha (Japan)	*Lactobacilluscasei* Shirota (*Lactobacillus* *casei* YIT 9029)	*Lacticaseibacillusparacasei* Shirota	LcS
Fonolla 2019 [50]	Biosearch life (Spain)	*Lactobacillus coryniformis* K8 CECT5711	*Loigolactobacillus coryniformis* K8 CECT5711	LK8
Lefevre 2015 [52]	Lesaffre (France)	*Bacillus subtilis* CU1 (*Bacillus subtilis* CNCM I-2745)	N/A	BsCU
Wang 2018 [54]	Culturelle (Denmark)	*Lactobacillus rhamnosus* GG (*L. rhamnosus* ATCC 53103)	*Lacticaseibacillus rhamnosus* GG	LGG

^1^ (Zheng et al., 2020); N/A: not applicable.

**Table 4:** Description of eight clinical studies on the effects of fermented drinks with probiotics and probiotic food supplements against acute upper respiratory tract infections in older people.

Reference	Study Design	Setting/Timeline	Enrolments and Allocation	Intervention		
				Active	Control	Duration
**Supplementation with Fermented Milk or Yoghurt with Probiotic Strains**						
Turchet 2003 [55]	randomized, open label, placebo- controlled pilot study.	Referential medical centers, Cordenons (Italy). Winter season. Time unspecified.	360 healthy free-living individuals over 60 years of age. 180 in treatment group (mean age: 67.1 ± 6.0 years), 180 in control group (mean age: 69.3 ± 5.6 years).	100 mL bottle of fermented dairy drink with *Lacticaseibacillus paracasei subsp. paracasei* CNCM I-1518 (10^8^ cfu/mL) and yoghurt cultures.	None taken.	1 bottle per day for 3 weeks
Makino 2010 [39]	randomized, double blind, placebo- controlled two-arm study.	Funagata (Japan): 13 March 2005–7 May 2005.	57 healthy elderly individuals.29 in treatment group, 28 in control group (mean age: 74.5 years).	90 g yoghurt fermented with *Lactobacillus delbrueckii* subsp. *bulgaricus* OLL1073R-1 (2.0–3.5 × 10^8^ cfu/g) and yoghurt culture *Streptococcus thermophilus* OLS3059 (6.3–8.8 × 10^8^ cfu/g). Extracellular polysaccharides 36.5–68.0 mg/kg	100 mL milk.	1 portion daily for 8 weeks.
		Arita (Japan): 14 November 2006–5 February 2007.	85 healthy elderly individuals. 42 in treatment group, 43 in control group (mean age: 67.7 years).			
Guillemard 2010 [38]	multicenter, randomized, double blind, placebo-controlled parallel group study.	125 general practitioners in 25 centers (France). November 2006 to May 2007, including follow-up period.	1072 free-living elderly individuals. 537 in treatment group (mean age: 76 years), 535 in control group (mean age: 76 years).	Fermented dairy drink (Actimel) containing *Lacticaseibacillus paracasei subsp. paracasei* CNCM I-1518 (10^10^ cfu/100 g) and yoghurt cultures: *Streptococcus thermophilus* and *Lactobacillus delbrueckii* subsp. *bulgaricus* (10^10^ cfu g).	Non- fermented, acidified, sweetened, flavored dairy drink	2 drinks daily for 3 months (84 days) and 1-month follow-up phase.
Fujita 2013 [51]	Multicenter, randomized, double blind, placebo-controlled study.	Four day-care facilities in Tokyo (Japan). December to June 2009, including observation period.	154 users of day-care facilities. (mean age: 83.2 ± 9.1 years). 78 in treatment group, 76 in control group.	80 mL fermented milk containing lactic acid bacteria, high-fructose corn syrup, sugar, skimmed milk powder and 4 × 10^10^ cfu *Lacticaseibacillus casei* Shirota (LcS).	Fermented drink containing the same as above except probiotic (LcS).	1 drink daily for 4 months and 3-months observation period.
Puyenbroeck 2012 [53]	Multicenter, randomized, double blind, placebo-controlled study.	53 nursing homes in Antwerp region (Belgium). October 2007 to April 2008.	554 nursing home residents (mean age: 84.17 years). 282 in treatment group, 272 in control group.	Fermented milk with *Lacticaseibacillus* *casei* Shirota (LcS) (6.5 × 10^9^ cfu).	Similar drink without probiotic.	Twice daily for 176 days.
**Supplementation with food supplements containing probiotics**						
Fonolla 2019 [50]	Multicenter, randomized, double blind, placebo-controlled trial.	Five nursing homes in Granada (Spain). October/November to April 2016, including observation period.	84 nursing home residents, older than 65 years. (mean age: 81.76 ± 7.2 years). 38 in treatment group, 46 in control group.	Capsule with 3 × 10^7^ *Loigolactobacillus coryniformis* K8 CECT5711 cfu in matrix of maltodextrin	Capsule with 300 mg maltodextrin.	1 capsule daily 2 weeks before influenza vaccination. 5-month follow-up period.
Lefevre 2015 [52]	randomized, double blind, placebo-controlled, parallel arms study.	Nantes area (France). Winter season 2010–2011, including observation period	100 free-living subjects, aged 60–74 years, 50 in treatment group (mean age: 63.3 (2.8) years). 50 in control group (mean age: 63.0 (2.4) years)	Capsule with *Bacillus subtilis* CU1 (2 × 10^9^) and excipients: maltodextrin DE14, dicalcic phosphate, magnesium stearate, colloidal silica.	Capsule with excipient mix.	10 days intermittently, alternating 18-day break, repeated 4 times.
Wang 2018 [54]	multicenter, pilot, double-blind, randomized, placebo-controlled study.	14 nursing homes in Ontario (Canada). March 2013 to July 20, including observation period.	196 nursing home residents aged 65 and older. 100 in treatment group (mean age: 85.2 ± 7.1 years). 96 in control group, (mean age: 85.9 ± 7.0 years)	Capsule with *Lacticaseibacillus rhamnosus* GG (10^9^)	Capsule with calcium carbonate.	2 capsules daily for 6 months.

**Table 5:** Reported outcomes of eight clinical studies on the effects of fermented drinks with probiotics and probiotic food supplements against acute upper respiratory tract infections in older people.

Reference	Probiotic	Incidence of Respiratory Tract Disease or Winter Infections	Duration of Respiratory Tract Disease	Immunological Parameters	Other Reported Outcomes
Turchet 2003	LpCNCM	No difference in incidence of winter infections (including influenza, gastrointestinal disease, ear-nose-throat pathology, bacterial broncho-pneumopathy) between groups	Significantly lower duration of all pathologies in treatment group (7.0 ± 3.2 days; n = 180) vs. control (8.7 ± 3.7 days; n = 180) (*p* = 0.024)	Not reported	Significantly lower maximal temperature (38.3 ± 0.5 °C) in treatment group vs. control (38.5 ± 0.6 °C) (*p* = 0.01).
Makino 2010	LbR-1	Significantly lower risk of catching the common cold (OR 0.39; *p* = 0.019) in treatment group vs. control. The risk was about 2.6 times lower.	Duration of URTI not reported	Significantly higher increase of natural killer cell activity in treatment group vs. control (*p* = 0.028).	/
Guillemard 2010	LpCNCM	Significantly lower incidence of URTI in treatment group vs. control (*p* = 0.004).	Significantly lower cumulative duration of URTI in treatment group vs. control (*p* = 0.004). The median episode duration was 1–1.5 days shorter for treatment group vs. control.	Immunological parameters were comparable between the two groups.	Significantly lower duration of CID episodes and the cumulative duration of CID in treatment group vs. control (*p* = 0.008 and 0.009, respectively). No statistically significant difference between groups regarding cumulative number of CID.
Fujita 2013	LcS	Total number of acute URTI events/total days of observation was lower in treatment group (0.0066) vs. control (0.0372), but not statistically significant (*p* = 0.64).	Statistically significant lower mean duration of infection per infection event was shorter in treatment group (3.71 ± 2.18 days) vs. control (5.40 ± 3.86 days), (*p* = 0.037).	Not reported.	Total symptom score/total days of observation (LcS: 0.0412, placebo: 0.0372, *p* = 0.89) was higher in treatment group vs. control, but not statistically significant.
Puyenbroeck 2012	LcS	No significant difference between treatment group vs. control for number of participants with respiratory symptoms (*p* = 0.325).	No significant difference between treatment group vs. control for the number of days with respiratory symptoms (*p* = 0.342).	Not reported.	No significant differences between groups regarding influenza vaccination response.
Fonolla 2019	LcK8	Incidences of symptoms usually associated with respiratory infections were lower in the treatment group vs. control, although the differences reached significance only for sore throat. Incidence of local respiratory symptoms (sore throat, cough and/or nasal congestion) was approximately 48% lower in treatment group vs. control (*p* = 0.007).	Duration of URTI not reported	No significant differences in immunological parameters ^1^ were found in both groups.	The odds of seroconversion for at least one of the antigens of the vaccine was 4.94 times higher in the treatment group vs. control (*p* = 0.036). The odds of analgesic consumption were significantly lower in the treatment group (more than 6 times) vs. control (OR = 0.151; 95% CI 0.022–0.641; *p* = 0.021).
Lefevre 2015	BsCU1	In the subset of 44 subjects, the frequency of respiratory infections was significantly lower in the treatment group vs. control (*p* = 0.0323): a mean number of 0.6 (0.7) respiratory infections was observed in the probiotic group vs. 1.1 (0.9) in the placebo group	Duration of URTI not reported	In a subset of 44 subjects, IFN-γ concentrations significantly increased in the treatment group (*p* = 0.0090). No significant differences in concentrations of cytokines ^2^.	No statistically significant difference between treatment group vs. control in mean duration, intensity and frequency of CID (*p* = 0.2361, *p* = 0.7400, *p* = 0.3290, respectively).
Wang 2018	LGG	No statistically significant differences in confirmed viral respiratory infections, influenza-like illness, hospitalization over pneumonia or other measured outcome between groups.	Duration of URTI not reported	Not reported	/

LpCNCM (Lacticaseibacillus casei DN-114 001); Lb1073 (Lactobacillus delbrueckii subsp. bulgaricus OLL1073R-1); LcS (Lacticaseibacillus casei Shirota); LcK8 (Loigolactobacillus coryniformis K8 CECT5711); BsCU (Bacillus subtilis CU1); LGG (Limosilactobacillus rhamnosus GG); ^1^ immunoglobins (IgA, IgG), cytokines (IL-4, IL-10, TNF-α); ^2^ (IL-1b, IL-4, IL-6, IL-8, IL-10, IL-12p70, IgA and TNF-α).

## Data Availability

No original data produced.
